# Quantitative evaluation of respiratory motion‐induced blurring in expiratory‐phase CBCT for pancreatic SBRT

**DOI:** 10.1002/acm2.70729

**Published:** 2026-07-31

**Authors:** Airi Nukushina, Ryota Yamada, Takaaki Yoshimura, Norio Katoh, Kentaro Nishioka, Hiroshi Tamura, Masataka Kitagawa, Keiji Kobashi, Takahiro Kanehira, Hidefumi Aoyama, Takayuki Hashimoto

**Affiliations:** ^1^ Graduate School of Biomedical Science and Engineering Hokkaido University Sapporo Japan; ^2^ Department of Radiation Technology Hokkaido University Hospital Sapporo Japan; ^3^ Faculty of Health Sciences Hokkaido University Sapporo Japan; ^4^ Global Center for Biomedical Science and Engineering Faculty of Medicine Hokkaido University Sapporo Japan; ^5^ Department of Medical Physics Hokkaido University Hospital Sapporo Japan; ^6^ Department of Radiation Oncology Faculty of Medicine Hokkaido University Sapporo Japan; ^7^ Department of Radiation Oncology Hokkaido University Hospital Sapporo Japan

**Keywords:** CBCT, pancreatic cancer, respiratory motion, SBRT

## Abstract

**Background:**

Cone‐beam computed tomography (CBCT) is widely used in image‐guided radiotherapy because it reflects the patient's anatomy immediately before treatment. However, CBCT images of the abdominal organs, including the pancreas, are often degraded by respiratory motion.

**Purpose:**

To address this limitation, this study quantitatively evaluated expiratory‐phase CBCT (EP‐CBCT), an acquisition method designed to reduce respiratory motion‐induced blurring during stereotactic body radiotherapy (SBRT) for pancreatic cancer.

**Methods:**

This retrospective analysis included 10 patients with pancreatic cancer undergoing SBRT at our institution. Daily CT and EP‐CBCT scans were acquired on each treatment day. EP‐CBCT images were obtained with repeated end‐exhalation breath‐holds to collect projection data during the expiratory phase. Because of feasibility limitations in routine clinical practice, a direct intra‐patient comparison with conventional free‐breathing CBCT was not performed. Image quality was evaluated using edge response width (ERW), structural similarity index (SSIM), and a blinded subjective assessment based on mean opinion scores (MOS). A supplementary retrospective comparison with a historical four‐dimensional CBCT (4D‐CBCT) dataset was also performed.

**Results:**

Differences in ERW between daily CT and EP‐CBCT (ΔERW) were evaluated using equivalence testing with a predefined equivalence margin of ± 1 mm. The mean ERW (98% confidence interval) was 2.04 mm (1.95–2.13) for EP‐CBCT and 1.73 mm (1.66–1.79) for daily CT. The mean ΔERW was −0.31 mm (−0.40 to −0.23 mm). Equivalence testing demonstrated that EP‐CBCT was statistically equivalent to daily CT (*p *< 0.01). The mean SSIM value was 0.83, indicating high structural similarity between EP‐CBCT and daily CT. Subjective image quality assessment showed significantly higher scores for daily CT than for EP‐CBCT, whereas EP‐CBCT consistently outperformed historical 4D‐CBCT, with good interobserver agreement.

**Conclusion:**

These findings suggest that EP‐CBCT can reduce respiratory motion‐induced blurring to a degree comparable to that of daily CT, supporting its potential utility for anatomical assessment immediately before irradiation in patients undergoing pancreatic SBRT.

## 1 INTRODUCTION

Pancreatic cancer remains a highly lethal malignancy, with both incidence and mortality rates that continue to increase worldwide. In 2022, an estimated 510 566 new cases and 467 005 deaths were reported globally, making pancreatic cancer the sixth leading cause of cancer‐related deaths.^1^ Most patients present with advanced, unresectable disease at diagnosis, which substantially limits curative treatment options and contributes to poor prognosis.

In recent years, stereotactic body radiotherapy (SBRT) has emerged as a promising treatment option for locally advanced, unresectable pancreatic cancer because of its potential to improve local control.^2^ SBRT delivers highly focused radiation from multiple directions to a small target volume, maximizing tumor control while minimizing radiation exposure to surrounding normal tissues.^3^

However, the pancreas is anatomically adjacent to radiosensitive gastrointestinal organs, particularly the stomach and duodenum, and excessive radiation exposure to these structures can lead to severe gastrointestinal toxicity (≥ grade 3).^4^ Therefore, minimizing radiation exposure to organs at risk (OARs) is essential.

The complex motion of the pancreas and surrounding organs further complicates treatment. The pancreas can shift in position and deform due to respiration, peristalsis, and variations in gastrointestinal volume.^5^ These motion patterns vary considerably among patients.^6^ Thus, an accurate assessment of the spatial and temporal relationships between the tumor and OARs is critical for safe and effective SBRT.

Recent advances in image‐guided radiation therapy have enabled highly precise irradiation while accounting for tumor motion.^7^ Among these techniques, cone‐beam computed tomography (CBCT) is routinely used clinically for patient positioning.^8^ However, unlike conventional computed tomography (CT), which can be acquired during a single breath‐hold, CBCT on a C‐arm linear accelerator requires a relatively long acquisition period, typically approximately 1 min for a full 360° rotation. Consequently, CBCT is typically acquired during free breathing, resulting in respiratory motion–induced blurring of both the tumor and surrounding OARs.

Our institution uses CBCT not only for patient setup but also as an essential reference for selecting the most appropriate treatment plan in offline adaptive radiotherapy (ART) for pancreatic SBRT. This workflow requires clear visualization of both the target and OARs to ensure an accurate assessment of the target position relative to the OARs immediately before irradiation. However, conventional free‐breathing CBCT (FB‐CBCT) images are often degraded by respiratory motion,^9^ highlighting the need for improved image acquisition methods. To address this limitation and ensure safer, more effective radiotherapy, we introduced expiratory‐phase CBCT (EP‐CBCT) during SBRT for pancreatic cancer, using repeated end‐exhalation breath‐holds. Although advanced image‐guidance systems such as MR‐guided radiotherapy and CT‐on‐rails provide superior soft‐tissue visualization and adaptive capabilities, their implementation is often restricted by substantial capital costs, specialized infrastructure, and dedicated workflow requirements. Therefore, practical image‐guidance strategies using standard linac‐mounted CBCT systems remain clinically valuable, particularly in institutions without access to these advanced technologies. This study aimed to assess the impact of respiration‐induced internal motion on EP‐CBCT image quality. Although positional reproducibility under breath‐hold conditions has been examined previously, quantitative evaluation of respiratory motion‐induced image blurring on EP‐CBCT remains limited.^10^

## 2 MATERIALS AND METHODS

### 2.1 Patients and study setting

This retrospective study enrolled patients with pancreatic cancer who underwent SBRT at our institution between November 2021 and June 2023. During the study period, 12 patients underwent pancreatic SBRT. CT and EP‐CBCT were performed on each treatment day in all cases. Two patients were excluded from the analysis: one because of unavailable imaging data, and the other because of a different EP‐CBCT acquisition protocol (half‐rotation). Accordingly, 10 patients were included in the analysis. Patient characteristics are summarized in Table 1. This study was approved by the institutional review board (registration number 025–0263), which waived the requirement for informed consent because of the retrospective design and anonymization of all patient data. Additionally, all patients were given the opportunity to opt out of the study.

**TABLE 1:** Patient characteristics.

Characteristics		Values	Median	Min	–	Max
Patients		10				
	Male	7				
	Female	3				
	Age		80	50	–	85
	Height [cm]		160.2	152.0	–	169.6
	Weight [kg]		63.2	46.3	–	79.1
	BMI [kg/m^2^]		24.5	20.0	–	29.4
Tumor location	Head	7				
	Body	1				
	Body and tail	1				
	Tail	1				
Fiducial marker	VISICOIL	5				
	Gold Anchor	5				

Abbreviation: BMI, body mass index.

At our institution, pancreatic SBRT was delivered using an offline adaptive plan library approach. Before treatment, three treatment plans with different dose levels were generated based on the planning CT. The prescribed dose was 40 Gy in 5 fractions. All plans were designed so that 50% of planning target volume and the gross tumor volume received the prescription dose. For the plan library approach, two alternative plans were generated by scaling the monitor units of the reference treatment plan. On each treatment day, a daily CT was acquired to assess anatomy and evaluate candidate treatment plans according to the gastrointestinal dose constraints. Immediately before irradiation, EP‐CBCT was acquired to verify patient anatomy and positioning, and the final treatment plan was confirmed prior to treatment delivery. Real‐time tumor‐tracking radiation therapy (RTRT) was used for all treatments. All treatments were delivered using a TrueBeam linear accelerator (Varian Medical Systems, Palo Alto, CA, USA) with a 6‐MV flattening filter‐free photon beam. Real‐time tumor tracking was performed in the end‐expiratory phase under free‐breathing conditions using fluoroscopic imaging with SyncTraX software (Shimadzu Co., Kyoto, Japan).

RTRT is a highly accurate irradiation method achieved by implanting fiducial gold markers near the tumor and monitoring them in real time using two orthogonal X‐ray fluoroscopes.^11^ Fiducial gold markers were implanted in all patients. VISICOIL (IBA Dosimetry, Schwarzenbruck, Germany) was used for patients treated before February 2023, and Gold Anchor (Naslund Medical AB, Huddinge, Sweden) was used thereafter.

### 2.2 Image acquisition

**FIGURE 1:**
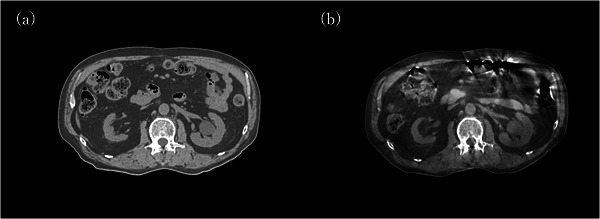
Representative images of daily CT and EP‐CBCT. (a)Daily CT, (b) EP‐CBCT. CT, computed tomography; EP‐CBCT, expiratory‐phase cone‐beam computed tomography.

Representative daily CT and EP‐CBCT scans are shown in Figure 1a,b, respectively.

#### 2.2.1 CT

All patients underwent planning and daily CT imaging in the supine position. Both arms were raised using a Posirest (CIVCO Medical Solutions, Coralville, IA, USA). Patients were immobilized using an ESFORM vacuum cushion (600 × 1500 mm) (Engineering System, Nagano, Japan) during CT scanning. CT images were acquired using a SOMATOM Confidence scanner (Siemens Healthineers, Forchheim, Germany) with breath‐holding at end expiration. The CT acquisition parameters were as follows: tube voltage, 120 kV; tube current–time product, 300 mAs; slice thickness, 2 mm; matrix size, 512 × 512; and field of view, 500 mm.

#### 2.2.2 EP‐CBCT

The EP‐CBCT acquisition parameters were as follows: tube voltage, 140 kV; tube current–time product, 540 mAs; slice thickness, 2 mm; matrix size, 512 × 512; gantry rotation speed, 6°/s; 900 projections; and an irradiation time of 60 s. During image acquisition, the patient's respiratory waveform was monitored using a Real‐time Position Management system (Varian Medical Systems, Palo Alto, CA, USA). At the start of each acquisition, the end‐expiratory phase was set as the zero‐amplitude reference, and the operators confirmed that breath‐holding was performed at this point. The patients were instructed to control their breathing during acquisition.

**FIGURE 2:**
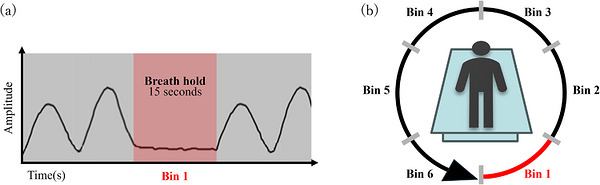
EP‐CBCT acquisition. (a) Respiratory waveform showing repeated end‐exhalation breath‐holds of approximately 15 s, during which the CBCT projection data were acquired. (b) Data acquired during the end‐exhalation breath‐hold phase (Bin1) were accumulated over multiple acquisitions to complete the 360° EP‐CBCT dataset. EP‐CBCT, expiratory‐phase cone‐beam computed tomography; CBCT, cone‐beam computed tomography.

EP‐CBCT was performed under repeated end‐expiratory breath‐holds lasting approximately 15 s. After each acquisition, the patients resumed breathing freely. This procedure was repeated until the completion of a full 360° rotation. The EP‐CBCT images were reconstructed using only the end‐expiratory phase projections (Figure 2). Because respiratory patterns varied among patients, breathing was rehearsed before imaging to estimate the breath‐hold duration at end expiration. Images were reconstructed using iterative CBCT (Varian Medical Systems, Palo Alto, CA, USA) with a medium noise‐suppression level and a smooth reconstruction filter.

#### 2.2.3 4D‐CBCT (supplementary analysis)

For the supplementary analysis, a historical four‐dimensional CBCT (4D‐CBCT) dataset from one patient with pancreatic SBRT was retrospectively analyzed. The acquisition parameters were as follows: tube voltage, 125 kV; tube current, 40 mA; pulse width, 20 ms; frame rate, 7 frames/s; gantry rotation speed, 3°/s; total tube current–time product, 672 mAs; and slice thickness, 2 mm. The reconstructed end‐expiratory (50%) phase images were used for the supplementary analysis.

All imaging data were acquired under end‐expiratory conditions, which served as the common reference state for image acquisition and comparison.

## 3 EVALUATION METRICS

### 3.1 Objective image quality assessment

#### 3.1.1 Edge Response Width (ERW)

To quantify the image blurring caused by respiratory motion, we used ERW, a metric that reflects the sharpness of boundary region.^12^ This approach was selected because respiratory motion predominantly affects the superior–inferior (SI) direction.^13^ Coronal images from daily CT and EP‐CBCT were analyzed.

**FIGURE 3:**
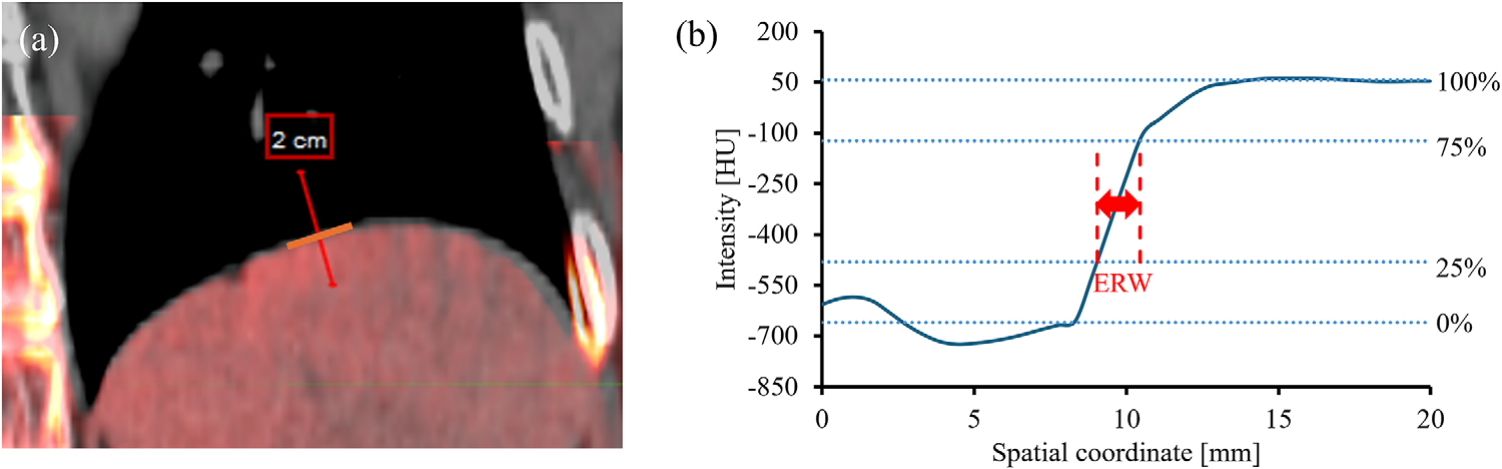
Method for ERW determination.

ERW was defined as the spatial distance over which image intensity increased from 25% to 75% along an approximately 2 cm linear profile drawn across a tissue boundary (Figure 3). The mean CT values from the inner and outer 5 mm regions were defined as 100% (inside the tissue) and 0% (outside the tissue), respectively. This procedure reduced the influence of local anatomical heterogeneity and voxel‐level intensity fluctuations. A smaller ERW reflects a steeper CT intensity gradient, indicating sharper boundaries and less respiratory motion‐induced blur.

Because consistent ERW measurement at pancreatic tumor sites was not feasible with CBCT owing to limited soft‐tissue contrast, alternative high‐contrast anatomical interfaces were evaluated, including the superior and inferior borders of both kidneys and the right and left diaphragmatic surfaces. These structures are affected by respiratory motion and are typically included in the CBCT field of view, providing adequate spatial coverage for ERW measurement.

All measurements were performed using MIM Maestro version 7.4.3 (MIM Software Inc., Cleveland, OH, USA). Linear intensity profiles were obtained perpendicular to the boundaries, and profiles corresponding to identical spatial coordinates in the CT and CBCT images were extracted after rigid registration.

We analyzed 543 measurement points from daily CT and EP‐CBCT images across all treatment fractions in 10 patients. Three measurement points were sampled for each image at each anatomical site. Measurement points were excluded when reliable ERW estimation was not feasible because of (1) insufficient edge separation caused by proximity to adjacent anatomical structures, (2) discontinuity or incomplete visibility of the boundary within the imaging field, or (3) severe image artifacts that prevented reliable extraction of intensity profiles. The number of measurement points varied because of these exclusions.

To assess the differences in ERW between daily CT and EP‐CBCT, ΔERW was calculated using the following equation:

(1) $$\begin{array}{c} \Delta ERW=ERW_{EP-CBCT}-ERW_{\mathrm{daily}\;CT} \end{array}$$

- Rigidly registered daily CT and EP‐CBCT images with linear profiles were drawn across the tissue boundaries.
- The corresponding intensity profiles along the line are shown in panel (a). The ERW is defined as the distance between the spatial coordinates at which the HU values reach 25% and 75%.

ERW, edge response width; CT, computed tomography; EP‐CBCT, expiratory‐phase cone‐beam computed tomography

#### 3.1.2 Structural similarity index measure (SSIM)

To evaluate overall image similarity between daily CT and EP‐CBCT images, the SSIM was calculated after image registration.^14^ SSIM is a full‐reference image‐quality metric that evaluates similarity in luminance, contrast, and structural information between two images. SSIM values range from 0 to 1, with higher values indicating greater structural similarity. SSIM was calculated for each paired daily CT and EP‐CBCT image set, and the mean value was used for the analysis.

### 3.2 Subjective image quality assessment

Subjective image quality was independently evaluated by two experienced board‐certified radiation oncologists (TH, 25 years; and KN, 18 years, respectively). Daily CT, EP‐CBCT, and conventional 4D‐CBCT images were anonymized and presented in random order without information about image modality or acquisition technique.

Three image quality attributes were assessed using a 5‐point Likert scale: (1) overall image quality, (2) boundary visibility, and (3) plan‐selection feasibility. Overall image quality reflected the overall diagnostic quality. Boundary visibility assessed the clarity of anatomical interfaces.^15^ Plan‐selection feasibility reflected the reviewer's confidence in selecting an appropriate treatment plan based on the image. For all categories, a score of 1 indicated very poor quality or confidence, whereas a score of 5 indicated excellent quality or confidence.

The mean score from the two reviewers was defined as the mean opinion score (MOS). Interobserver agreement was evaluated using Cohen's weighted kappa and intraclass correlation coefficients (ICCs).

For qualitative reference, six conventional 4D‐CBCT datasets acquired from different patients and treatment fractions were retrospectively evaluated using the same ERW and subjective assessment methods. These datasets were included for descriptive comparison only and were not incorporated into the primary statistical analyses.

### 3.3 Statistical analysis

We compared the ERW values between daily CT and EP‐CBCT images using a paired equivalence test based on the two one‐sided tests (TOST) procedure.^16^ Given the 2 mm slice thickness of daily CT and EP‐CBCT, we set the equivalence margin at ± 1 mm, corresponding to half of the slice thickness. This threshold was considered physically reasonable because CT slice thickness influences longitudinal spatial resolution and partial‐volume effects, and differences within half the slice thickness are likely to fall within the uncertainty associated with image sampling and partial‐volume effects.^17^, ^18^ Furthermore, ERW represents the 25%–75% intensity transition width across an anatomical boundary and serves as a quantitative metric of edge sharpness rather than a direct measure of target localization accuracy.^12^ Therefore, the ± 1 mm equivalence margin was intended to evaluate equivalence in image sharpness rather than localization accuracy. This margin is also consistent with the millimeter‐level geometric accuracy generally required for image‐guided SBRT.^19^ For the equivalence analysis using the TOST procedure, a significance level of *p* < 0.01 was adopted.

For subjective image quality assessment, differences in MOS between daily CT and EP‐CBCT were evaluated using the paired Wilcoxon signed‐rank test. Statistical significance was defined as *p* < 0.05. Interobserver reliability was assessed using Cohen's weighted kappa and the intraclass correlation coefficient based on a two‐way mixed‐effects, single‐measure, absolute‐agreement model [ICC (3,1)], because the same two radiation oncologists evaluated all images and were treated as fixed observers in this study. Weighted kappa values were interpreted according to the benchmarks proposed by Landis and Koch, with values of 0.61–0.80 indicating substantial agreement and values > 0.80 indicating almost perfect agreement. ICC values < 0.50 were considered poor, 0.50–0.75 moderate, 0.75–0.90 good, and > 0.90 excellent reliability.^20^, ^21^

Statistical analyses were performed using the JMP Student Edition version 18 (SAS Institute, Cary, NC, USA).

## 4 RESULTS

### 4.1 EP‐CBCT acquisition

Among the 10 patients included in this study, the mean total EP‐CBCT acquisition time, including the free‐breathing intervals, was 299.5 ± 125.9 s (range, 146–762 s). Image acquisition required a mean of 7.1 ± 1.3 end‐expiratory breath‐holds (range, 5–10).

### 4.2 ERW analysis

Across all measurement points (*n* = 543), the mean ERW (98% confidence interval [CI]) was 2.04 mm (1.95–2.13) for EP‐CBCT and 1.73 mm (1.66–1.79) for daily CT. The mean ΔERW (98% CI) was −0.31 mm (−0.40 to −0.23 mm).

Equivalence testing demonstrated that the ERW values for EP‐CBCT were statistically equivalent to those for daily CT (*p *< 0.01), as the 98% CI of the mean ΔERW fell entirely within the predefined equivalence margin (± 1 mm).

**FIGURE 4:**
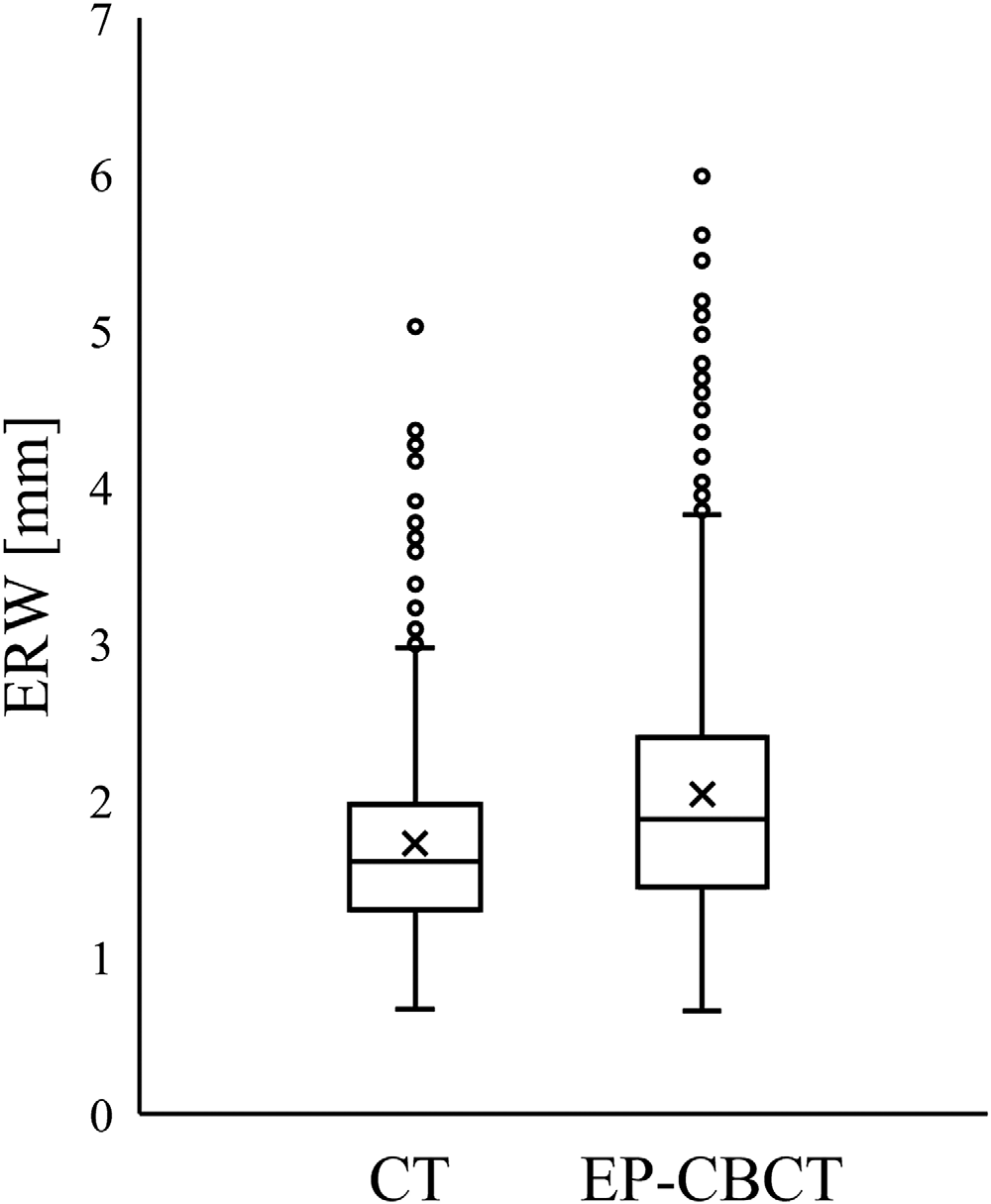
Box‐and‐whisker plot of ERW for daily CT and EP‐CBCT. ERW, edge response width; CT, computed tomography; EP‐CBCT, expiratory‐phase cone‐beam computed tomography.

The distribution of ERW values is shown in Figure 4, and site‐specific results are provided in Table 2.

**TABLE 2:** ERW values at each anatomical site on daily CT and EP‐CBCT.

Region	*N*	ERW (CT) [mm] Ave (98% CI)	ERW (EP‐CBCT) [mm] Ave (98% CI)	ΔERW [mm]
All	543	1.73 (1.66 to 1.79)	2.04 (1.95 to 2.13)	−0.31 (−0.40 to −0.23)
RKS	105	1.86 (1.71 to 2.00)	2.35 (2.15 to 2.54)	−0.49 (−0.67 to −0.30)
RKI	135	1.57 (1.44 to 1.69)	1.92 (1.75 to 2.10)	−0.36 (−0.52 to −0.20)
LKS	129	1.85 (1.72 to 1.97)	2.08 (1.90 to 2.25)	−0.23 (−0.40 to −0.07)
LKI	111	1.67 (1.53 to 1.81)	1.88 (1.69 to 2.07)	−0.22 (−0.39 to −0.04)
DR	24	1.74 (1.45 to 2.04)	1.96 (1.55 to 2.37)	−0.22 (−0.60 to 0.16)
DL	39	1.68 (1.45 to 1.91)	2.00 (1.68 to 2.32)	−0.32 (−0.62 to −0.02)

Abbreviations: CI, confidence interval; CT, computed tomography; DL, left diaphragm; DR, right diaphragm; EP‐CBCT, expiratory‐phase cone‐beam computed tomography; ERW, edge response width; LKI, left kidney inferior; LKS, left kidney superior; RKI, right kidney inferior; RKS, right kidney superior; ΔERW, difference between daily CT and EP‐CBCT.

### 4.3 Additional image quality assessment

The mean SSIM between daily CT and EP‐CBCT was 0.830 ± 0.066, indicating high structural similarity between the two image sets.

The results of the subjective image quality assessment are summarized in Table 3. The MOSs for EP‐CBCT were 2.30 for overall image quality, 3.02 for boundary visibility, and 2.79 for plan‐selection feasibility, whereas the corresponding scores for daily CT were 4.83, 4.85, and 4.88, respectively. All three subjective image quality metrics were significantly higher for daily CT than for EP‐CBCT (Wilcoxon signed‐rank test, all *p* < 0.001). For qualitative reference, conventional 4D‐CBCT datasets yielded lower MOS values of 1.00, 1.42, and 1.00 for the corresponding categories.

**TABLE 3:** Subjective image quality assessment and interobserver agreement.

	MOS (95% CI)						
Category	CT	EP‐CBCT	4D‐CBCT*	*p* value†	Weighted *k*	ICC(3,1)	Mean difference
Overall image quality	4.83 (4.76–4.90)	2.30 (2.18–2.42)	1.00 (N/A)	< 0.001	0.878	0.901	0.331
Boundary visibility	4.85 (4.78–4.92)	3.02 (2.88–3.16)	1.42 (1.20–1.63)	< 0.001	0.766	0.86	0.576
Plan‐selection feasibility	4.88 (4.82–4.95)	2.79 (2.64–2.95)	1.00 (N/A)	< 0.001	0.812	0.873	0.508

Abbreviations: 4D‐CBCT, four‐dimensional cone‐beam computed tomography; CI, confidence interval; CT, computed tomography; EP‐CBCT, expiratory‐phase cone‐beam computed tomography; ICC, intraclass correlation coefficient; κ, Cohen's weighted kappa; MOS, mean opinion score.

*Descriptive evaluation only. Images were acquired from different patients and treatment fractions and were not included in statistical comparisons.

†Wilcoxon signed‐rank test comparing CT and EP‐CBCT.

Interobserver agreement is also summarized in Table 3. Weighted kappa values were 0.878, 0.766, and 0.812 for overall image quality, boundary visibility, and plan‐selection feasibility, respectively, and the corresponding ICC (3,1) values were 0.901, 0.860, and 0.873, indicating good agreement between the two radiation oncologists.

**FIGURE 5:**
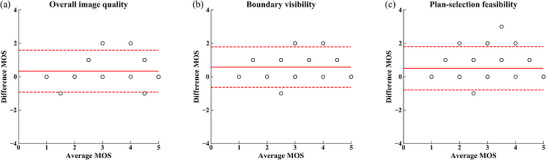
Bland–Altman plots for interobserver agreement in subjective image quality assessment. (a) Overall image quality, (b) boundary visibility, and (c) plan‐selection feasibility. The solid line represents the mean difference (TH − KN), and the dashed lines indicate the 95% limits of agreement (mean difference ± 1.96 SD).

Bland–Altman analysis was performed to assess systematic differences between the two reviewers. The mean differences (TH − KN) were 0.331, 0.576, and 0.508 points for overall image quality, boundary visibility, and plan‐selection feasibility, respectively. The corresponding Bland–Altman plots are shown in Figure 5.

### 4.4 Supplementary comparison with conventional 4D‐CBCT

As a supplementary analysis, we retrospectively analyzed a historical 4D‐CBCT dataset acquired from a patient treated with pancreatic SBRT. ERW measurements were performed at the right and left diaphragmatic interfaces using the same methodology as described above, with three measurement points sampled for each interface. A total of 36 measurement points obtained from five treatment fractions and one rehearsal scan were analyzed. The mean ERW measured on the 50% phase (end‐exhalation) image was 2.88 mm, which was greater than the diaphragmatic mean ERW values observed for EP‐CBCT (1.98 mm) and daily CT (1.71 mm).

## 5 DISCUSSION

In this study, we quantitatively evaluated the effect of respiratory motion on EP‐CBCT image quality in SBRT for pancreatic cancer using ERW. CBCT images are generally affected by respiratory motion; however, the proposed EP‐CBCT technique acquires projection data only during exhalation. The mean ΔERW between EP‐CBCT and daily CT was ‐0.31 mm, and the ERW values were statistically equivalent, indicating that EP‐CBCT effectively suppresses the effects of respiratory motion.

One possible factor contributing to an increased ERW is misalignment of exhalation phases associated with multiple breath‐holds. Whereas daily CT scans are acquired during a single breath‐hold, EP‐CBCT requires multiple breath‐holds. This variability can lead to excessive or insufficient exhalation and residual image blur. This variability may be mitigated by appropriate respiratory guidance. Although we observed no significant differences in ERW among the 10 patients in this analysis, future studies could explore patient‐specific strategies to improve image quality further.

Breath‐hold duration is another important factor affecting image quality. During the first few seconds of breath‐holding, organs primarily move in the SI direction, with maximum displacement at end‐inhalation and minimal displacement at end‐exhalation.^22^ Therefore, maintaining and extending breath‐holding at the end of exhalation may further improve image quality. In this study, breath‐holding lasted for approximately 15 seconds. However, previous research on patients with pancreatic cancer demonstrated that all participants were able to maintain 20 seconds of breath‐holding using appropriate devices.^10^ These findings suggest that extending the breath‐hold duration through proper respiratory training can reduce the number of repeated acquisitions and improve phase consistency.

Respiratory motion remains a major challenge for CBCT‐based image guidance in pancreatic radiotherapy because it degrades image quality and obscures anatomical structures. Several imaging methods have therefore been developed to reduce respiratory motion‐induced image degradation during treatment imaging. Among these approaches, 4D‐CBCT reconstructs images from a specific respiratory phase, typically at end‐exhalation. However, this approach can result in lower signal‐to‐noise ratios, prolonged acquisition times, and increased imaging dose because only a subset of the acquired projection data is used for image reconstruction.^23^ To provide supplementary evidence regarding respiratory motion‐induced image blurring and baseline respiratory imaging approaches, we retrospectively evaluated a historical 4D‐CBCT dataset acquired from a patient treated with pancreatic SBRT. In this analysis, only diaphragmatic interfaces were evaluated because image noise and reduced soft‐tissue contrast precluded reliable identification of renal boundaries on the 4D‐CBCT images. Our analysis showed that the mean ERW measured on the 50% phase (end‐exhalation) image was greater than the corresponding diaphragmatic ERW values observed for EP‐CBCT and daily CT. Because the 50% phase image is reconstructed from projection data assigned to a finite respiratory phase bin during free breathing, residual intra‐phase motion blurring may remain. In contrast, EP‐CBCT is acquired during repeated end‐expiratory breath‐holds, which may further reduce motion‐related image degradation.

Objective ERW findings were further supported by supplementary image quality analyses. Although peak signal‐to‐noise ratio (PSNR) is widely used for image quality evaluation, it was not adopted in the present study because daily CT and EP‐CBCT were acquired at different time points and may differ in bowel gas, gastrointestinal contents, breath‐hold reproducibility, and organ deformation. Because PSNR is highly sensitive to voxel‐wise intensity differences, these anatomical variations could substantially influence the measured values and obscure differences attributable to image quality itself. Instead, SSIM was used as a complementary metric because it incorporates local luminance, contrast, and structural information and is less sensitive to localized anatomical differences. The mean SSIM of 0.83 indicated high structural similarity between daily CT and EP‐CBCT.

Subjective image quality assessment further supported the objective findings. Although EP‐CBCT received significantly lower MOSs than daily CT for all evaluation categories, it consistently achieved higher subjective scores than conventional 4D‐CBCT. Furthermore, good interobserver agreement was observed across all evaluation categories, with weighted kappa values ranging from 0.766 to 0.878 and ICC (3,1) values ranging from 0.860 to 0.901. Bland–Altman analysis demonstrated a consistent tendency for one reviewer to assign slightly higher scores than the other; however, the overall agreement remained good. These findings suggest that EP‐CBCT may provide improved subjective image quality compared with the historical 4D‐CBCT datasets evaluated in this supplementary analysis.

Although these findings demonstrate improved image quality with EP‐CBCT, they should not be interpreted as evidence that EP‐CBCT alone is sufficient for online ART. Online ART requires not only high‐quality volumetric imaging but also accurate target delineation, deformable image registration, treatment plan re‐optimization, and dosimetric verification, which were beyond the scope of the present study. Taken together, these objective and subjective findings consistently demonstrate that EP‐CBCT reduces respiratory motion‐induced image blurring while preserving anatomical structures with sufficient clarity for image‐guidance purposes.

Beyond respiratory phase‐resolved CBCT, MRI provides clear soft‐tissue visualization without additional radiation exposure for image guidance.^24^ However, its use is limited to specialized centers because of substantial capital investment, specialized infrastructure, and dedicated clinical workflows.^25^ Similarly, advanced in‐room imaging systems such as CT‐on‐rails require additional equipment and integration with the treatment workflow.^26^ In contrast, EP‐CBCT can be implemented using a standard linac‐mounted CBCT system without additional hardware and may provide a practical and cost‐effective approach to respiratory motion management for institutions without access to these advanced technologies. More recently, CBCT platforms such as HyperSight (Varian Medical Systems, Palo Alto, CA, USA) have substantially shortened image acquisition time compared with conventional CBCT, thereby improving workflow efficiency.^27^ However, the achievable acquisition time still depends on the treatment platform and imaging protocol, and these technologies are not yet widely available. In contrast, the EP‐CBCT technique evaluated in this study provides a practical alternative for institutions where ultrafast CBCT systems are unavailable.

This study has several limitations. First, the sample size was small. Because of the limited annual patient volume at our institution, further increasing the sample size within the study period was not feasible. However, previous studies focused on image quality assessment have typically included relatively small cohorts of approximately 10–20 patients.^9^, ^28^, ^29^ Future studies involving larger cohorts and multicenter collaborations are warranted to confirm the generalizability and reproducibility of these findings.

Second, direct visualization of the pancreas and its boundaries on CBCT is inherently challenging because of poor soft‐tissue contrast.^30^ Because respiratory motion is a global factor that degrades edge sharpness across all abdominal structures,^9^ the ERW was evaluated at high‐contrast anatomical interfaces, such as the diaphragm and kidneys, rather than at the tumor boundary itself. The diaphragm has been used as a representative respiratory motion interface in 4D imaging studies to quantify motion‐related blurring,^31^ and the kidneys have been used as reproducible upper abdominal landmarks in prior studies evaluating respiratory motion‐corrected CBCT.^28^ Although these sites do not represent the direct motion of the pancreatic tumor, they serve as robust, objective indicators of overall image sharpness and respiratory motion‐induced blurring.

Third, a direct comparison with conventional FB‐CBCT was not possible because FB‐CBCT is not routinely acquired for pancreatic SBRT at our institution. To partly address this limitation, we performed an additional retrospective analysis using a historical 4D‐CBCT dataset. However, this analysis was limited to a single patient and did not represent a direct intra‐patient comparison with conventional FB‐CBCT. Therefore, further validation using larger datasets and other respiratory imaging approaches is warranted. In the future, the use of ERW may facilitate comparisons between EP‐CBCT and other modalities and institutions through multicenter collaborations.

Fourth, we did not perform a dosimetric evaluation of the cumulative dose from treatment plan modifications because of technical challenges, including the limited accuracy of deformable image registration.^32^ Because the primary focus of this study was the image quality of EP‐CBCT, we did not perform a dosimetric analysis. Future studies should incorporate dosimetric evaluations into their research design.

In conclusion, EP‐CBCT can provide sufficiently clear images immediately before irradiation, reflecting the patient's internal anatomy while reducing respiration‐induced motion blur. Our analysis showed that the difference between daily CT and EP‐CBCT was only 0.31 mm, which is small compared with the CT slice thickness of 2 mm, indicating that the anatomical structures remained clearly visible for clinical purposes. These findings suggest that EP‐CBCT achieves image quality comparable to daily CT and may therefore support more confident offline treatment plan selection immediately before irradiation.

## AUTHOR CONTRIBUTIONS

Airi Nukushina and Ryota Yamada contributed equally to this study. Airi Nukushina, Hiroshi Tamura and Takaaki Yoshimura collected and analyzed the data and drafted the manuscript. Ryota Yamada contributed to data collection and data analysis. Norio Katoh and Takahiro Kanehira contributed to the clinical methodology. Masataka Kitagawa and Keiji Kobashi contributed to the statistical analysis. Kentaro Nishioka, Hidefumi Aoyama, and Takayuki Hashimoto supervised the study and revised the manuscript. All authors reviewed and approved the final manuscript.

## CONFLICT OF INTEREST STATEMENT

The authors have no relevant conflicts of interest to disclose.

## FUNDING INFORMATION

The authors have nothing to report.

## ETHICS STATEMENT

This study was approved by the ethics committee of Hokkaido University Hospital (IRB: 025–0263). The requirement for written informed consent was waived, and all patients were given the opportunity to opt out.

## Data Availability

Research data are not shared due to privacy restrictions.
